# Prognosis of Hyponatremia in Elderly Patients With Fragility Fractures

**DOI:** 10.14740/jocmr1984w

**Published:** 2014-10-16

**Authors:** Kirsten Cumming, Stephen McKenzie, Graeme E. Hoyle, James D. Hutchison, Roy L. Soiza

**Affiliations:** a School of Medicine & Dentistry, University of Aberdeen, Aberdeen, UK; b Department of Medicine for the Elderly, NHS Grampian, Aberdeen Royal Infirmary, Aberdeen, UK

**Keywords:** Hyponatremia, Sodium, Geriatrics, Fractures, Prognosis, Length of admission

## Abstract

**Background:**

Hyponatremia (serum sodium < 135 mmol/L) is the commonest electrolyte imbalance encountered in clinical practice. It is associated with multiple poor clinical outcomes including increased length of hospital stay, institutionalization and mortality. Prevalence of hyponatremia is higher in frail patient groups, and elderly patients with fragility fractures (EPFF) are particularly susceptible. This study aimed to establish the impact of hyponatremia on total length of inpatient stay (TLOS), need for inpatient rehabilitation and mortality in EPFF.

**Methods:**

Prospective observational study of consenting adults aged ≥ 65 years admitted with a fragility fracture to a university hospital between January 7, and April 4, 2013. Demographic and clinical data, length of hospital stay, discharge destination and any participant deaths were recorded. Prevalence of hyponatremia on admission and incidence of cases developing in hospital were reported. Basic demographic data and serum sodium results were included in multivariate linear regression models for TLOS. Difference in mortality rate and proportion of individuals discharged to inpatient rehabilitation between the hyponatremic and normonatremic group were tested using Chi-squared and Fisher’s exact tests. Unadjusted odds ratios (ORs) and 95% confidence intervals (CIs) were also calculated.

**Results:**

Of 212 cases, 127 (60%) EPFF were recruited (mean age 79 years, 78% female). Of those not recruited, 66 had incapacity to consent and 19 refused participation. Thirty-three cases of hyponatremia were identified; point prevalence on admission was 13.4% and a further 12.6% developed hyponatremia during admission. There were no statistically significant differences in patient characteristics between the hyponatremic and normonatremic group. Hyponatremic participants had a 66.7% increased time from admission to surgery (P = 0.014) and a 51.5% increased length of index hospital stay (P = 0.006). Factors independently associated with increased TLOS were age (P = 0.03) and drop in sodium during admission (P < 0.001). Mortality rate and proportion of participants discharged to inpatient rehabilitation were higher in the hyponatremic group (OR 4.2 (95% CI: 0.9 - 19.8) and 2.2 (95% CI: 0.9 - 5.1), respectively), but figures did not reach statistical significance.

**Conclusions:**

Hyponatremia is highly prevalent in EPFF, seen in 33/127 cases (26%), and is associated with increased length of index hospital stay. Drop in serum sodium during admission was independently associated with increased TLOS.

## Introduction

Hyponatremia (serum sodium < 135 mmol/L) is the commonest electrolyte imbalance encountered in clinical practice. It is associated with multiple poor clinical outcomes including increased mortality, institutionalization and length of hospital stay [1-3]. Acute and severe (serum sodium < 125 mmol/L, developing < 24 h) hyponatremia may present with obvious neurological symptoms which can result in well recognized, serious complications, e.g. seizure, coma and in almost 50% of cases, death [2]. However, most cases of hyponatremia are mild and chronic (serum sodium 130 - 134 mmol/L, developing > 24 h) and are typically devoid of the obvious neurological symptoms seen in acute severe hyponatremia [4]. Consequently, chronic mild hyponatremia is frequently considered asymptomatic despite being strongly associated with major geriatric conditions and multi-organ pathological changes. These include abnormal gait patterns, cognitive impairment, bone demineralization, falls and fractures [5-8]. In the absence of obvious neurological symptoms, chronic mild hyponatremia is at risk of being overlooked and may subsequently cause any of the aforementioned complications. Published series have consistently shown that any degree of hyponatremia, even mild, can entail significant clinical consequences [6, 8-10]. Previous studies have found that age, co-morbidities, rate of onset, severity and etiology of hyponatremia are determinants of the clinical complications encountered on patient-specific bases [1-3, 9, 10]. Whether hyponatremia is an independent predictor of patient outcomes or a marker of disease severity remains to be established. Nevertheless, hyponatremia may be amenable to treatment and is often preventable, so its association with poor clinical outcomes is important.

Prevalence of hyponatremia is known to increase in frail patient groups, such as elderly, hospitalized and peri-operative patients with a fracture [5, 11-13]. Elderly patients with fragility fractures (EPFF) are particularly susceptible to hyponatremia due to degenerate physiology, multiple co-morbidities, polypharmacy, hospitalization, peri-operative fluid restrictions and homeostatic stress from fracture and subsequent surgical interventions [4]. EPFF are also at higher risk of complications of hyponatremia, making this group of particular clinical importance. In EPFF, hyponatremia may be a contributing factor in fracture etiology and may worsen prognosis after fracture in this already frail patient group [5]. Despite this, to our knowledge, there are no studies specifically investigating the prognostic implications of hyponatremia after fragility fracture in older people.

This study aims to investigate the impact of hyponatremia on key clinical outcomes in EPFF, total length of inpatient stay (TLOS), need for ongoing inpatient rehabilitation and death.

## Materials and Methods

We conducted a prospective observational study of consenting adults aged 65 years and above admitted with a fragility fracture to Aberdeen Royal Infirmary (ARI), a university teaching hospital, from January 7, to April 4, 2013. Fragility fractures were defined as those occurring either without trauma or due to low energy trauma, equivalent to a fall from standing height or less than 1 m. Participants were recruited from ARI acute orthopedic trauma wards (OTW) and geriatric assessment unit (GAU). Following advice from the Research Ethics Committee, adults with incapacity were excluded from recruitment. Capacity to consent was assessed by the attending clinical team in accordance with the Adults with Incapacity (Scotland) Act 2000.

The following demographic and clinical data were recorded for each participant: age, gender, type of residence prior to admission, co-morbidities, medication, fracture site, surgical intervention and time to surgical intervention from admission and length of stay in OTW or GAU (index hospital admission). Details were obtained from patient interview and medical and nursing notes. Medication data were obtained by reconciling patient and/or carer histories, and primary and secondary care records. Pre-admission, admission and subsequent inpatient serum sodium measurements were obtained, where available, from laboratory computer systems. The laboratory is the only public health service laboratory covering the study population, so it was unlikely that patients had more recent investigations elsewhere. The maximum drop in serum sodium during each participant’s stay was calculated by subtracting the lowest sodium level recorded during in-patient stay from the sodium level on admission. Similarly, the drop in serum sodium between pre-admission and lowest in-patient sodium level was calculated. Outcome measures investigated were TLOS, discharge destination and death. Date of discharge from OTW or GAU and discharge destination were recorded for each participant. For participants initially discharged to intermediate care facilities (e.g. rehabilitation centers), discharge date and final discharge destination were obtained using the electronic patient management system, TrakCare. This allowed TLOS to be calculated (days between admission and final discharge from inpatient care). Any participant deaths and date and cause of death were recorded.

Cases of hyponatremia were defined as any serum sodium measurement < 135 mmol/L. The prevalence of hyponatremia on admission and the incidence of cases developing in hospital were calculated. The proportion of participants with known hyponatremia prior to their fracture was calculated by obtaining the last available serum sodium for each patient prior to their hospital admission. Prevalence of hyponatremia at discharge was calculated according to the last available serum sodium measurement prior to discharge from OTW or GAU.

Independent T tests or Mann-Whitney U tests were used, as appropriate, to describe hyponatremic and normonatremic groups. Pearson Chi-squared and Fisher’s exact tests were calculated to compare proportions across groups. Spearman’s rank correlation coefficient was employed to assess correlation between TLOS and sodium-related variables. Multivariate linear regression analysis identified independent effects on TLOS after controlling for potential confounders. Variables included in the model were selected based on Pearson’s product correlation coefficients which were calculated for the following covariates: age, gender, number of co-morbidities and sodium-related variables. Difference in mortality rate and proportion of individuals discharged to inpatient rehabilitation between the hyponatremic and normonatremic group were tested using Chi-squared and Fisher’s exact tests. Unadjusted odds ratios (ORs) and 95% confidence intervals (CIs) were also calculated. Statistical Package for Social Sciences (SPSS) version 20.0 was used to perform all analyses. Statistical significance was assumed where P < 0.05.

Ethical approval was obtained from the Scotland A Research Ethics Committee (Ref 12/SS/0209). The original protocol submitted to the Committee had provision for the inclusion of adults with incapacity to consent but the Committee’s approval was dependent on their exclusion. The Committee’s recommendations were fully incorporated into the final study design and final approval was obtained from the Committee Scientific Advisor.

## Results

There were 212 EPFF identified and 127 were recruited into the study. Those not recruited had incapacity to consent (N = 66) or declined participation (N = 19). There were no statistically significant differences between participants and those who were eligible but declined participation. Compared to participants, adults with incapacity were on average 7 years older (P < 0.001) and had different proportions of fracture site, with hip fractures composing 71.2% (N = 47) of fractures compared to 52.8% (N = 67) in participants (P = 0.017). All volunteers had at least one serum sodium measurement but sodium measurements were available in only 44% of the total patient-days in hospital.

Of those recruited (N = 127), mean age was 79 years, 78% were female (N = 97) and 52.8% (N = 67) had hip fractures. Thirty-three cases of hyponatremia were identified during the study (26% of cases). The characteristics and outcomes of the 127 participants are detailed in Tables 1 and 2, categorized according to natremic state. Apart from sodium measurements, there were no statistically significant differences in characteristics between the hyponatremic and normonatremic group. A similar proportion of individuals received surgical intervention in the two groups (P = 0.631). However, mean length of time from admission to surgery was higher in the hyponatremic group (3 days) compared to the normonatremic group (1.8 days), a 66.7% increase (P = 0.014). Hyponatremic participants also had a 3.5 day longer mean length of index hospital stay (10.3 days), compared to normonatremic participants (6.8 days), a 51.5% increase (P = 0.006).

**Table 1:** Participant Characteristics

Participant characteristic	Normonatremic participants	Hyponatremic participants	P value
Number (N)	94	33	
Age (years)			
Average (± SD)	78 (± 8)	81 (± 7)	0.083
Range	65 - 97	69 - 98	
Female sex % (N)	76.6 (72)	81.8 (27)	0.078
Previous residence % (N)			
Own home	85.1 (80)	78.8 (26)	0.39
Sheltered housing	14.9 (14)	12.1 (4)	
Nursing/care home	0	6.1 (2)	
Care of the elderly medicine	0	3 (1)	
Number of co-morbidities (N)			
Mean (± SD)	4 (± 2)	5 (± 3)	0.222
Range	0 - 9	0 - 11	
Number of medications (N)			
Mean (± SD)	5 (± 4)	6 (± 3)	0.294
Range	0 - 15	0 - 15	
Fracture site % (N)			0.115
Hip	50 (47)	57.6 (19)	
Hip and upper limb	1.1 (1)	0	
Other lower limb*	24.5 (23)	15.2 (5)	
Other lower limb* and upper limb	0	3 (1)	
Upper limb	21.3 (20)	15.2 (5)	
Pelvic and upper limb	0	0	
Pelvic	3.2 (3)	3 (1)	
Vertebrae	0	6.1 (2)	
Time from fracture to hospital admission (days)			
Median	0	0	0.581
Interquartile range	0 - 1	0 - 1.5	
Serum sodium prior to admission (mmol/L)			
Mean (± SD)	140.4 (2.41)	136.2 (± 5)	< 0.001
Range	133 - 145	127 - 144	
Serum sodium on admission (mmol/L)			
Mean (± SD)	139 (± 2.4)	134 (± 4)	< 0.001
Range	135 - 146	123 - 141	
Prevalence of hyponatremia % (N)			
Prior to admission**	3.2 (3)	30.3 (10)	< 0.001
On admission	0	51.5 (17)	< 0.001

*Other lower limb: all lower limb fractures other than hip fractures. **According to last serum sodium measurement available prior to admission.

**Table 2:** Participant Outcomes

Characteristic	Normonatremic participants	Hyponatremic participants	P value
Number (N)	94	33	
Surgical management %(N)	76.6 (72)	81.8 (27)	0.631
Time to surgery from admission (days)			
Mean (± SD)	1.8 (± 7)	3 (± 3)	0.014
Range	0 - 7	0 - 10	
Incidence of hyponatremia developing in hospital % (N)	0	48.5 (16)	< 0.001
Patient deaths % (N)	4.5 (3)	12.9 (4)	0.07
Length of stay in OTW or GAU (days)			
Mean (± SD)	6.8 (± 4.97)	10.3 (± 8.4)	0.006
Range	0 - 27	1 - 45	
Discharge destination % (N)			0.51
Previous residence	29.8 (28)	21.2 (7)	
Relative’s residence	4.3 (4)	0	
Orthopedic rehabilitation	55.3 (52)	72.7 (24)	
Care of the elderly medicine	3.2 (3)	3 (1)	
Other medical speciality	2.1 (2)	0	
Died in acute hospital or data not obtained*	5.3 (5)	3 (1)	

OTW: orthopedic trauma ward; GAU: geriatric assessment unit. *Data not obtained: patients still acute hospital inpatients.

Prevalence of known hyponatremia prior to hospital admission was 10.2% (N = 13). Point prevalence of hyponatremia upon admission was 13.4% (N = 17) and incidence of cases developing in hospital was 12.6% (N = 16). Prevalence of hyponatremia at discharge was 19% (N = 24). Hyponatremia was predominantly mild (Na 130 - 134 mmol/L) (N = 25, 75.8%). Seven patients (5.6%) died during their total inpatient admission.

Spearman’s analysis showed a negative correlation between TLOS and inpatient sodium change (p_s_ = -0.476, P < 0.001), pre-admission to lowest inpatient sodium change (p_s_ = -0.376, P < 0.001) and lowest inpatient sodium measurement (p_s_ = -0.316, P = 0.003). Multivariate linear regression analysis showed factors independently associated with increased TLOS were increasing age (P = 0.03) and a larger drop in serum sodium during inpatient stay (P < 0.001). The adjusted R^2^ was 0.247, indicating that 24.7% of the variability in length of admission was explained by the factors in the model. Pre-admission serum sodium, lowest inpatient serum sodium and change between pre-admission sodium to lowest inpatient sodium were excluded from the model because of colinearity with inpatient sodium change. All other confounders apart from patient age were excluded as correlations with TLOS were not statistically significant. No significant interaction terms between the measured variables were identified. A larger proportion of hyponatremic individuals needed ongoing inpatient rehabilitation after index hospital admission. In the hyponatremic group 72.7% (N = 24) of participants were discharged to inpatient rehabilitation compared to 55.3% (N = 52) of the normonatremic group (P = 0.099) (OR 2.2, 95% CI: 0.9 - 5.1). There was a higher incidence of inpatient death in the hyponatremia group, 12.9% (N = 4) compared to 4.5% (N = 3) in the normonatremic group (P = 0.07). The OR for death amongst hyponatremic patients was 4.2 (95% CI: 0.9 - 19.8).

## Discussion

The findings demonstrate that hyponatremia is highly prevalent in EPFF, occurring in 33/127 cases (26%). Compared to normonatremics, hyponatremic individuals had a 66.7% longer time from admission to surgery and a 51.5% increased length of index hospital admission. Inpatient drop in serum sodium and age were independent predictors of TLOS. There was a higher proportion of discharges to inpatient rehabilitation and inpatient deaths in the hyponatremic group but these results did not reach statistical significance.

Consistent with previous literature, the majority of hyponatremia in this study was mild and therefore at risk of being unidentified or ignored. There were no statistically significant differences in characteristics between hyponatremic and normonatremic participants in our study sample. However, other series have found that hyponatremia is associated with increased age and higher co-morbidity score [1, 3, 8, 9]. Differences in findings may be attributable to variation in study sample population, small sample size or our exclusion of adults with incapacity. The mean length of time from admission to surgery was 66.7% longer in hyponatremic participants (3 days) compared to normonatremic participants (1.8 days) (P = 0.014). This delay to surgery is a potential risk factor for hyponatremia, perhaps due to dehydration or inappropriate fluid and electrolyte management, and suggests that patients with admission or pre-operative hyponatremia may have more complicated co-morbidities or fractures and therefore require a pre-operative preparatory period. This finding is important considering that some studies have shown an association between increased length of time to surgery and morbidity and mortality in fragility fractures of the hip [14]. We also found that hyponatremic participants had a 3.5 day longer mean length of index hospital stay, compared to normonatremic participants, a 51.5% increase (P = 0.006). This finding is consistent with multiple reports that hyponatremia is associated with increased length of hospital stay in a variety of clinical settings including a recent study of hyponatremia in traumatic hip fractures [1, 3, 9, 15, 16]. Delayed discharge in hyponatremic patients may be caused by attempts to correct hyponatremia but this was clearly not the case in our study as hyponatremia was usually left untreated, indicated by the 19% prevalence of hyponatremia at discharge. More plausible alternative explanations are that hyponatremia is a marker of dyshomeostasis or co-morbidities which delay discharge, or directly cause subtle cognitive or functional impairments that complicate discharge. EPFF discharged with hyponatremia are at risk of poor clinical outcomes additional to those associated with fractures. Gankam Kengne et al reported that four of 18 EPFF with hyponatremia at discharge had recurrent falls and fractures [5].

Increased age (P = 0.03) and a larger drop in serum sodium during inpatient stay (P < 0.001) were independently associated with increased TLOS. The modest adjusted R^2^ for the regression model, 0.247, reflects that a number of important predictors were not included in the model, potentially fracture site and pre-admission residence. This supports previous reports that those who develop hyponatremia in hospital or who experience worsening hyponatremia throughout hospital admission fare worse than those hyponatremic on admission [1, 3, 9]. Although a number of co-morbidities were used in this model, a more detailed analysis of morbidity, including severity and chronicity, as well as pre-admission level of dependence may have been of greater utility. An association between inpatient serum sodium drop and both increased length of hospital stay and loss of independence has previously been found in acute geriatric admissions [1]. Length of hospital stay has also been shown to increase with severity of hyponatremia and age in elderly and very elderly people [17]. Similarly, Wald et al found that the association between increased TLOS and hyponatremia strengthened with severity of hyponatremia [9]. Gill et al also reported that inpatient drop in serum sodium, rather than absolute admission sodium measurement, was associated with a higher mortality rate [3]. The consistent finding that drop in serum sodium during hospital admission is associated with adverse outcomes suggests that caution in preventing or minimizing hyponatremia in hospital could improve prognosis. In our study of predominantly mild hyponatremia, the findings related to TLOS emphasize that even in its mildest form, hyponatremia is associated with worse prognosis and therefore should not be considered asymptomatic.

A larger proportion of hyponatremic individuals needed inpatient rehabilitation after index hospital admission, although differences did not reach statistical significance (OR 2.2, 95% CI: 0.9 - 5.1). However, need for rehabilitation is largely determined by fracture site and level of dependence prior to fracture which were not accounted for in our analysis. Moreover, accurate assessment of level of dependence and need for ongoing inpatient care is difficult in the immediate post-fracture period, especially when comparing individuals with different fracture sites. Long-term follow-up of these patients (i.e. at 6 months) may have produced more representative findings. Previous work has shown that hyponatremia in older people is associated with institutionalization and loss of independence, with inpatient drop in serum sodium being an independent predictor [1]. A large study of over 50,000 general hospitalized adults also found that hyponatremia was associated with discharge to short- or long-term care facilities and that the strength of this association increased with severity of hyponatremia [9]. Investigation of these “hard” end points, whilst of practical utility to patients and healthcare providers, does not assess for subtle degrees of impairment and loss of independence which may result from hyponatremia. Subsequently these could contribute to significant clinical outcomes such as falls, fractures and recurrent hospitalizations.

There was a higher incidence of inpatient death amongst hyponatremics compared to normonatremics but results did not reach statistical significance (OR 4.2, 95% CI: 0.9 - 19.8). The lack of statistical significance is mostly likely due to the small number of deaths encountered in this study (N = 7) which precluded any detailed analysis of mortality. Previous work has consistently shown that hyponatremia is an independent predictor of mortality [9, 16, 18, 19]. Other studies have also demonstrated that hyponatremia is associated with increased mortality [2, 3, 10]. A recent meta-analysis of moderate hyponatremia and mortality reported a 2.47 - 3.34 relative risk of mortality across a variety of clinical sub-groups and an inverse correlation between overall mortality and serum sodium [20]. Wald et al found that mild hyponatremia, defined as serum sodium 133 - 137, had a 1.51 adjusted OR for mortality and that the association between hyponatremia and mortality increased with severity of hyponatremia [9]. Waiker et al studying hospitalized adults similarly found that hyponatremia, even mild, is associated with an increased risk of mortality and that this association was more pronounced in patients admitted for orthopedic procedures [10]. Their study also demonstrated that this risk persisted at 5-year follow-up. Another important finding made by this group was that resolution of hyponatremia diminished the higher risk of mortality associated with hyponatremia. These findings are of particular relevance to EPFF, and the general geriatric population, in whom mild hyponatremia predominates [1]. Literature presents conflicting evidence regarding other factors implicated in the risk of mortality and hyponatremia, e.g. co-morbidities, age and recurrent hospital admissions [10, 19, 20]. However, patients who develop hyponatremia in hospital and who have a large inpatient drop in serum sodium appear to be at highest risk [3, 9]. This is important considering the 12.6% incidence of hyponatremia in this study which is significantly higher than previous accounts in EPFF, 2.6-5.5% [12, 21-23]. Clayton et al report that the risk of mortality increases with the number of underlying causes of hyponatremia [2]. This is of particular relevance to EPFF as three groups have shown that hyponatremia in older people is predominantly multi-factorial [2, 24, 25]. Also, two studies have shown that inappropriate and unsuccessful treatments of hyponatremia are associated with an even greater risk of mortality [18, 26].

Our study has some important limitations which should be acknowledged. The observational design did not permit the investigating team to request clinical tests. Consequently, serum sodium measurements were missing in 56% of total patient-days in hospital. This same limitation has previously been encountered by various other groups and confirms concerns that clinical awareness of hyponatremia is low, predisposing already frail patients to worsened clinical outcomes [5, 27, 28]. Since patients did not have daily serum sodium measurements, it is likely that our report may underestimate the true incidence of hyponatremia in EPFF. It is also possible that there were unidentified hyponatremic individuals within our normonatremic group. The small sample size of this single centered study was another principal limitation meaning that the results may be subject to selection and sampling bias. Moreover adults with incapacity, who compose a significant proportion of EPFF, were excluded from this study as dictated by the ethics committee. Given the independent association between hyponatremia and age, excluding this patient cohort at higher risk of hyponatremia (mean age 7 years older than participants), may compromise the generalizability of our findings. Hyponatremia may be a precipitant or contributing cause of incapacity in older people and EPFF and so inclusion of adults with incapacity in this area of research ought to be routine. A third limitation is the lack of detail involved in our comparison between the hyponatremic and normonatremic group and our assessment of confounding factors. A more detailed analysis including severity and chronicity of disease and level of dependence and residence prior to fracture may have produced more informative results. Alternatively, case-control methods could have been employed to eliminate confounding factors although this would require a much larger sample size. Lastly, outcomes measures investigated here were relatively rudimentary and did not assess any subtle degrees of impairment or loss of independence. However, it is unlikely that these factors could be accurately detected in the initial post-discharge period. Future studies should recruit a larger sample size over multiple centers, include adults with incapacity and investigate outcomes for a longer follow-up period including cognitive impairment, falls and recurrent fractures and hospital admissions.

Despite these limitations, to our knowledge, this is the first study to specifically investigate key clinical outcomes of hyponatremia in EPFF. This work adds to comprehensive evidence of poor prognosis associated with hyponatremia and suggests that mild hyponatremia is implicated in the prognosis of EPFF. Unlike other studies that have used discharge codes that can be unreliable, our study had a prospective design and availability of outcome data was complete.

In conclusion, this study demonstrates that hyponatremia is highly prevalent in EPFF and is associated with increased length of index hospital admission and increased length of time from admission to surgery. Inpatient drop in serum sodium level was independently associated with increased length of hospital stay. This adds to evidence that mild hyponatremia is not benign and is of important prognostic value. Whether hyponatremia directly contributes to poor clinical outcomes remains to be established and merits full investigation. Although it is currently unclear if targeting hyponatremia itself would improve patient outcomes such interventions have great potential to decrease falls and fractures in older people and generally improve prognosis in EPFF. Better still, prevention of hyponatremia would most likely safeguard pre-frail and frail older people from its associated poor clinical outcomes.

## References

[1] Chua M, Hoyle GE, Soiza RL (2007). Prognostic implications of hyponatremia in elderly hospitalized patients. Arch Gerontol Geriatr.

[2] Clayton JA, Le Jeune IR, Hall IP (2006). Severe hyponatraemia in medical in-patients: aetiology, assessment and outcome. QJM.

[3] Gill G, Huda B, Boyd A, Skagen K, Wile D, Watson I, van Heyningen C (2006). Characteristics and mortality of severe hyponatraemia-a hospital-based study. Clin Endocrinol (Oxf).

[4] Soiza RL, Hoyle GE, Chua MPW (2008). Electrolyte and salt disturbances in older people: causes, management and implications. Reviews in Clinical Gerontology.

[5] Gankam Kengne F, Andres C, Sattar L, Melot C, Decaux G (2008). Mild hyponatremia and risk of fracture in the ambulatory elderly. QJM.

[6] Renneboog B, Musch W, Vandemergel X, Manto MU, Decaux G (2006). Mild chronic hyponatremia is associated with falls, unsteadiness, and attention deficits. Am J Med.

[7] Verbalis JG, Barsony J, Sugimura Y, Tian Y, Adams DJ, Carter EA, Resnick HE (2010). Hyponatremia-induced osteoporosis. J Bone Miner Res.

[8] Hoorn EJ, Rivadeneira F, van Meurs JB, Ziere G, Stricker BH, Hofman A, Pols HA (2011). Mild hyponatremia as a risk factor for fractures: the Rotterdam Study. J Bone Miner Res.

[9] Wald R, Jaber BL, Price LL, Upadhyay A, Madias NE (2010). Impact of hospital-associated hyponatremia on selected outcomes. Arch Intern Med.

[10] Waikar SS, Mount DB, Curhan GC (2009). Mortality after hospitalization with mild, moderate, and severe hyponatremia. Am J Med.

[11] Soiza RL, Talbot HS (2011). Management of hyponatraemia in older people: old threats and new opportunities. Ther Adv Drug Saf.

[12] Tambe AA, Hill R, Livesley PJ (2003). Post-operative hyponatraemia in orthopaedic injury. Injury.

[13] Upadhyay A, Jaber BL, Madias NE (2006). Incidence and prevalence of hyponatremia. Am J Med.

[14] Leung F, Lau TW, Kwan K, Chow SP, Kung AW (2010). Does timing of surgery matter in fragility hip fractures?. Osteoporos Int.

[15] Rudge JE, Kim D (2014). New-onset hyponatraemia after surgery for traumatic hip fracture. Age Ageing.

[16] Tzoulis P, Bagkeris E, Bouloux PM (2014). A case-control study of hyponatraemia as an independent risk factor for inpatient mortality. Clin Endocrinol (Oxf).

[17] Turgutalp K, Ozhan O, Gok Oguz E, Horoz M, Camsari A, Yilmaz A, Kiykim A (2013). Clinical features, outcome and cost of hyponatremia-associated admission and hospitalization in elderly and very elderly patients: a single-center experience in Turkey. Int Urol Nephrol.

[18] Sturdik I, Adamcova M, Kollerova J, Koller T, Zelinkova Z, Payer J (2014). Hyponatraemia is an independent predictor of in-hospital mortality. Eur J Intern Med.

[19] Gefen S, Joffe E, Mayan H, Justo D (2014). Recurrent hospitalizations with moderate to severe hyponatremia in older adults and its associated mortality. Eur J Intern Med.

[20] Corona G, Giuliani C, Parenti G, Norello D, Verbalis JG, Forti G, Maggi M (2013). Moderate hyponatremia is associated with increased risk of mortality: evidence from a meta-analysis. PLoS One.

[21] Beloosesky Y, Hershkovitz A, Solovey B, Salai M, Weiss A (2011). Hip fracture post-operation dysnatremia and Na+-courses in different cognitive and functional patient groups. Arch Gerontol Geriatr.

[22] McPherson E, Dunsmuir RA (2002). Hyponatraemia in hip fracture patients. Scott Med J.

[23] Antonelli Incalzi R, Gemma A, Capparella O, Terranova L, Sanguinetti C, Carbonin PU (1993). Post-operative electrolyte imbalance: its incidence and prognostic implications for elderly orthopaedic patients. Age Ageing.

[24] Shapiro DS, Sonnenblick M, Galperin I, Melkonyan L, Munter G (2010). Severe hyponatraemia in elderly hospitalized patients: prevalence, aetiology and outcome. Intern Med J.

[25] Cumming K, Hoyle GE, Hutchison JD, Soiza RL (2014). Prevalence, incidence and etiology of hyponatremia in elderly patients with fragility fractures. PLoS One.

[26] Huda MS, Boyd A, Skagen K, Wile D, van Heyningen C, Watson I, Wong S (2006). Investigation and management of severe hyponatraemia in a hospital setting. Postgrad Med J.

[27] Saeed BO, Beaumont D, Handley GH, Weaver JU (2002). Severe hyponatraemia: investigation and management in a district general hospital. J Clin Pathol.

[28] Gosch M, Joosten-Gstrein B, Heppner HJ, Lechleitner M (2012). Hyponatremia in geriatric inhospital patients: effects on results of a comprehensive geriatric assessment. Gerontology.

